# Milk-alkali syndrome in a middle-aged woman after ingesting large doses of calcium carbonate: a case report

**DOI:** 10.4076/1757-1626-2-8198

**Published:** 2009-09-16

**Authors:** Mandy Grubb, Kumar Gaurav, Mukta Panda

**Affiliations:** 1 Department of Medicine, University of Tennessee, College of Medicine, 960 East Third Street, Suite 208, Chattanooga, TN 37403, USA; 2 Department of Internal Medicine, Division of Nephrology, University of Virginia, Charlottesville, Virginia, USA

## Abstract

**Introduction:**

Most cases of hypercalcaemia are secondary to malignancy or primary hyperparathyroidism. Here we report a case of hypercalcaemia that we have attributed to milk-alkali syndrome.

**Case presentation:**

A 51-year-old Caucasian woman with a past history of thyroidectomy and parathyroidectomy secondary to thyroid cancer developed an altered mental state and had an extremely high calcium concentration of 22.8 mg/dl (5.7 mmol/l). Investigations included work up for malignancy and hyperparathyroidism. However, the hypercalcaemia was attributed to ingestion of large doses of calcium carbonate, leading to milk-alkali syndrome. She was managed with intravenous fluids and withdrawal of calcium carbonate. The patient responded well to treatment, with normalization of the calcium concentration and clinical improvement.

**Conclusion:**

We present this case to remind clinicians of the importance of detailed history taking and of milk-alkali syndrome as a cause of hypercalcemia.

## Introduction

Most cases of hypercalcemia are secondary to malignancy or primary hyperparathyroidism [1]. Milk-alkali syndrome accounts for about 12% of cases and ranks third among the causes of hypercalcemia [2] This syndrome is caused by ingestion of large amounts of calcium along with absorbable alkali. The occurrence of milk-alkali syndrome had fallen with the use of non-alkali therapies for peptic ulcer disease [3]. However, recently the incidence has increased secondary to the use of calcium carbonate for the prevention and treatment of osteoporosis and hyperphosphataemia in patients with chronic kidney disease [4]. We report a case of milk-alkali syndrome secondary to ingestion of calcium carbonate.

## Case presentation

A 51-year-old Caucasian woman presented with malaise and generalized weakness for one week and altered mental status of unknown duration. She was awake, slow to respond, and disoriented, and a complete history was unobtainable. Her past medical history included thyroidectomy and radio-iodine therapy 12 years before for a Hurthle cell thyroid carcinoma. She had a 30 pack-year history of smoking. She had been taking levothyroxine for iatrogenic hypothyroidism and calcium for iatrogenic hypoparathyroidism, but had been lost to follow-up for 3 years before presentation. She was disoriented and in no acute distress. Her blood pressure was 149/77 mmHg, heart rate 59/min, and she had extremely dry mucous membranes, tenting of the skin, dry scaly skin, right eye anisocoria, and symmetrical hyporeflexia. The rest of the physical examination was normal. Her initial laboratory values are listed in Table 1. She had severe hypercalcaemia, mild anaemia, renal insufficiency, metabolic alkalosis, and hypothyroidism. The arterial blood gases confirmed metabolic alkalosis (pH 7.57, PaCO_2_ 47 mmHg (6.3 kPa), PaO_2_ 87 mmHg (11.6 kPa), HCO_3_ 43 mmol/l, 98% oxygen saturation on room air). A computerized tomographic scan of the brain was normal. She responded well to aggressive intravenous fluid administration and the calcium concentration fell slowly. She was also given levothyroxine and corticosteroids. Investigations included work-up for malignancy, hyperparathyroidism, vitamin D intoxication, calcium intoxication, and granulomatous diseases such as sarcoidosis. Because of the extremely high concentration of calcium, malignancy was high on the list of differential diagnoses. Protein electrophoresis, chest X-ray, colonoscopy, esophagoduodenoscopy, and breast examination were normal. Intact PTH and PTH-related peptide were undetectable in the serum. The serum concentration of 1,25(OH)_2_-Vitamin D was low at 7 pg/ml. After resolution of her confusion, she admitted to having consumed 7.2 g (175 mmol) of calcium per day and confirmed that her anisocoria was congenital. We therefore attributed her hypercalcaemia to milk-alkali syndrome, due to excessive intake of calcium carbonate.

**Table 1:** Pertinent laboratory values

Test	Patient Values	Reference Ranges
Calcium	22.8 mg/dL (5.7 mmol/L)	8.4-10 mg/dL
Albumin	4.6 g/dL (46 g/L)	3.4-4.8 g/dL
Total protein	8.5 g/dL (85 g/L)	6.4-8.3 g/dL
WBC count	10.6 × 109/L	4.8-10.8 × 109/L
Haemoglobin	10.9 g/dL (109 g/L)	12.0-16.0 g/dL
Haematocrit	31.4% (0.314)	37.0-47.0%
ESR	30 mm/hr	0-20 mm/hr
Iron saturation	10.10%	15-55%
Sodium	133 mmol/L	136-145 mmol/L
Potassium	2.6 mmol/L	3.5-5.1 mmol/L
Chloride	93 mmol/L	98-107 mmol/L
Bicarbonate	36 mmol/L	22-29 mmol/L
Blood urea nitrogen	48 mg/dL	9.8-20.1 mg/dL
Creatinine	2.1 mg/dL (186 µmol/L)	0.5-1.0 mg/dL
Magnesium	1.5 mg/dL (0.62 mmol/L)	1.7-2.2 mg/dL
Phosphate	2.9 mg/dL (0.94 mmol/L)	2.3-4.7 mg/dL
Alkaline phosphatase	137 U/L	40-150 U/L
TSH	89.8 mIU/mL	0.4-4.0 mIU/mL
Free T4	0.65 ug/dL (8.4 pmol/L)	5.20-12.5 ug/dL
CPK	473 U/L	29-168 U/L

Initial laboratory data showed severe hypercalcaemia, mild anaemia, renal insufficiency, metabolic alkalosis, and hypothyroidism.

## Discussion

Symptoms in hypercalcaemia may be secondary to the raised calcium concentration itself or to the underlying cause. Hypercalcaemia usually becomes symptomatic at a concentration of 12-14 mg/dl (3.0-3.5 mmol/l) [5]. The symptoms are predominantly related to the gastrointestinal and renal tracts and the nervous and musculoskeletal systems. These include abdominal pain, reduced appetite, nephrolithiasis, depressed mood, headache, confusion, lethargy, and muscle weakness [4]. Of all cases of hypercalcaemia 80-90% are secondary to malignancy or primary hyperparathyroidism, but many other disorders can cause hypercalcaemia [1]. A few that are important to remember include milk-alkali syndrome, vitamin D intoxication, and granulomatous diseases such as sarcoidosis. Milk-alkali syndrome is now the third most common cause and accounts for about 12% of cases [2]. The initial diagnostic work-up for hypercalcaemia includes serum PTH, phosphorus, 25-OH-cholecalciferol and 1,25 OH_2_-cholecalciferol, and urinary calcium.

Our patient had profound symptomatic hypercalcaemia, which caused lethargy, cognitive disturbance, and muscle weakness. Work-up for malignancy was negative and she gave a history of calcium and alkali ingestion. The clinical laboratory data were characteristic of milk-alkali syndrome and we excluded other causes of hypercalcemia [6].

In the early 20^th^ century, calcium-containing antacids and milk were used to treat peptic ulcer disease. Sometimes this therapy was associated with toxicity [6,7]. Hardt and Rivers identified the milk-alkali syndrome in 1923 and reported that it caused headache, nausea, vomiting, dizziness, musculoskeletal pains, and weakness [7]. Today, this syndrome continues to occur in patients who have ingested large amounts of calcium, in excess of 4 g/day with absorbable alkali, particularly as calcium carbonate [4,6]. The milk-alkali syndrome had become less frequent with the use of non-alkali therapies for peptic ulcer disease, but the incidence has recently increased, probably secondary to the use of calcium carbonate for osteoporosis and hyperphosphataemia in patients with chronic kidney disease [4].

**Figure 1:**
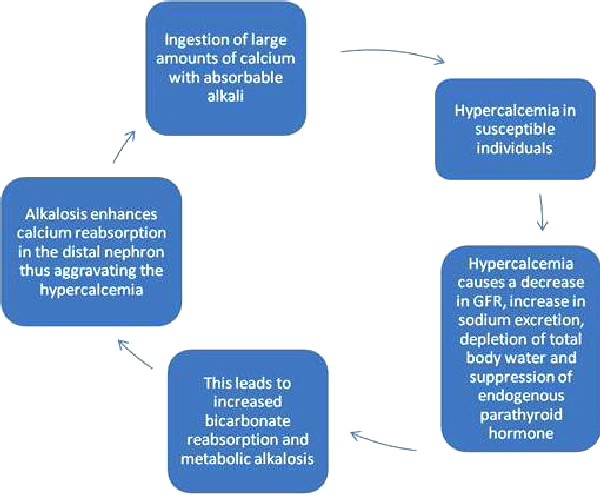
**Pathogenesis physiological explanation for high amounts of calcium intake as a cause of hypercalcemia**.

The pathophysiology of the milk-alkali syndrome is poorly understood. It is unclear why only some individuals are affected by excessive ingestion, but it has been suggested that they absorb more calcium than others [9]. The triad of milk-alkali syndrome includes hypercalcaemia, metabolic alkalosis, and renal insufficiency [8]. Hypercalcaemia causes reduced glomerular filtration rate, increased sodium excretion, depletion of total body water, and suppression of endogenous parathyroid hormone [9]. This leads to increased bicarbonate reabsorption and a metabolic alkalosis. Alkalosis enhances calcium reabsorption in the distal nephron, thus aggravating the hypercalcaemia [9]. This cycle continues as long as calcium and absorbable alkali are ingested (Figure 1).

The syndrome can present in three ways: acute, subacute, and chronic. The acute presentation includes weakness, myalgia, irritability, and apathy. Advanced or chronic milk-alkali syndrome, otherwise known as Burnett's syndrome, involves severe hypercalcaemia, phosphate retention, and irreversible renal insufficiency. This presentation can be accompanied by ectopic calcification. Subacute presentation, as in our patient, causes reversible renal insufficiency and usually improves over a period of weeks after withdrawal of oral calcium and alkali. Conservative treatment and supportive measures such as intravenous fluids should also be given [6].

## Conclusion

In patients with hypercalcaemia, after common causes such as hyperparathyroidism or malignancy have been ruled out, it is important to remember the milk-alkali syndrome as an increasingly common cause of hypercalcaemia. A detailed history, including the use of over-the-counter medications, should be taken in all patients [10].

## Consent

At our institution, the UT College of Medicine Chattanooga and Erlanger we have an Institutional Review Board (IRB) to review projects and ensure the protection of human subjects involved. The Chairman of the IRB is B W Ruffner, M.D. Our manuscript has been approved by our Institutional Review Board.

## Competing interests

The authors declare that they have no competing interests.

## Authors' contributions

MG, KG and MP analyzed and interpreted the patient data. MG and KG were major contributors in writing the manuscript. All authors read and approved the final manuscript.
